# Natural Deep Eutectic Solvents as Rust Removal Agents from Lithic and Cellulosic Substrates

**DOI:** 10.3390/molecules29030624

**Published:** 2024-01-28

**Authors:** Francesco Gabriele, Cinzia Casieri, Nicoletta Spreti

**Affiliations:** Department of Physical and Chemical Sciences, University of L’Aquila, I-67100 L’Aquila, Italy; francesco.gabriele@univaq.it (F.G.); cinzia.casieri@aquila.infn.it (C.C.)

**Keywords:** deep eutectic solvents, iron rust, lithic surface, cellulosic substrate, cleaning, reducing agent

## Abstract

The peculiar physicochemical features of deep eutectic solvents (DESs), in particular their tunability, make them ideal media for various applications. Despite their ability to solubilize metal oxides, their use as rust removers from valuable substrates has not yet been thoroughly investigated. In this study, we chose three known DESs, consisting of choline chloride and acetic, oxalic or citric acid for evaluating their ability to remove corrosion products from a cellulose-based material as linen fabric and two different lithotypes, as travertine and granite. The artificial staining was achieved by placing a rusty iron grid on their surfaces. The DESs were applied by means of cellulose poultice on the linen fabrics, while on the rusted stone surfaces with a cotton swab. Macro- and microscopic observations, colorimetry and SEM/EDS analysis were employed to ascertain the cleaning effectiveness and the absence of side effects on the samples after treatment. Oxalic acid-based DES was capable of removing rust stains from both stone and cellulose-based samples, while choline chloride/citric acid DES was effective only on stone specimens. The results suggest a new practical application of DESs for the elimination of rust from lithic and cellulosic substrates of precious and artistic value.

## 1. Introduction

Twenty years ago, the pioneering work of Abbott first reported that mixtures of choline chloride and urea had produced eutectics which were liquids at room temperature with unusual solvent properties [1]. Since then, numerous papers and reviews have been devoted to the development and characterization of several Deep Eutectic Solvents (DESs) in different fields of application [2,3,4,5,6]. In fact, thanks to their peculiar physicochemical properties, to their non-toxicity, biodegradability and biosustainability, and, above all, to their tunability, they can be designed to satisfy different and multiple requirements.

Although DESs are often presented as a new class of Ionic Liquids (ILs), they actually represent two different types of solvent [7]. DESs are mixtures of Lewis or Brønsted acids and bases, while ILs are composed of organic cations and organic or inorganic anions. Their preparation is different, since it is sufficient to mix, stir and heat the components of the DES, in a proper molar ratio, for obtaining a homogeneous liquid, due to the formation of an extensive network of hydrogen bonds. In this way, there is no waste generation and no purification phase is necessary. On the contrary, the synthesis and purification of an IL often requires the use of toxic organic solvents.

Many physical properties of DESs and ILs, such as low vapor pressure, thermal stability, low flammability, good electrical conductivity, high viscosity and an excellent ability to dissolve both organic and inorganic molecules, are similar, and both have the potential to be highly versatile solvents. Although ILs were long considered as “green solvents”, most of them appear not biodegradable, exhibit toxicity and are harmful for human health and the environment [8]. In an attempt to produce “truly green ILs”, new cation/anion combinations were developed, using renewable starting materials [9,10]; in fact, amino acids, sugars and choline represent some of the most used components for the production of a new generation of ILs.

DESs, developed as alternative solvents, appear to be less toxic than ILs [7], although considering the almost unlimited number of hydrogen-bond donors (HBD) and hydrogen-bond acceptors (HBA) that can compose them, their toxicity and cytotoxicity should be checked carefully before their use [11,12]. In addition, with the aim of preparing increasingly environmentally friendly solvents, the so-called NADES, composed of natural primary metabolites, such as organic acids, amino acids, and sugars, were developed and employed for several applications [13,14,15,16,17,18]. Nevertheless, suitable studies have to be performed also for the NADESs to effectively assess their biosustainability. In fact, some of them resulted non-toxic to a series of aquatic invertebrate species, but induced an intense growth of algae causing eutrophication issues [19].

In recent years, DESs have shown enormous potential for various applications thanks to their ability to solubilize compounds otherwise not soluble in classical organic solvents [20], such as separation and extraction processes [21,22,23,24], synthesis [25,26,27,28], biocatalysis [29,30], CO_2_ capture [31,32,33], drug delivery [34,35,36], to mention just a few recent reviews.

Another important application of DESs is in the field of the so-called solvometallurgy, also termed ionometallurgy, i.e., extraction of metals from ores, industrial processing residues and urban waste using non-aqueous solutions [37,38,39]. In fact, given the ability of DESs to solubilize metal oxides, they can be successfully used in place of very acidic or basic aqueous solutions, as required by hydrometallurgical techniques. In the literature, there are several papers dealing with the extraction of metal oxides by means of DESs constituted by a quaternary ammonium salt, mainly choline chloride and carboxylic acids [40,41], *p*-toluenesulfonic acid [42], sulphosalicylic acid [43] or chloroacetic acid [44]. Moreover, the effect of both acidity and complexing ability of the HBD was shown to have a significant effect on metal oxide dissolution and speciation, with important applications in their selective recovery and recycling processes [45]. Recently, an acidic DES based on choline chloride and diglycolic acid was successfully used for the cleaning of a rusted metal wire, showing also that the efficiency of DES depends on both temperature and removal time [46].

Regarding rust, the removal of its stains represents a topic of great concern, especially in the conservation of cultural heritage, due to the high thermodynamic stability and low solubility of the corrosion products of iron [47]. Different constituents of rust exist, and their distribution is affected by the environment in which the substrates are exposed. Under high humidity conditions, the formation of hydroxides or oxyhydroxides is favored, while in drier environments, ferric or mixed oxides are the predominant species. The physical and chemical approaches commonly used to clean lithic and cellulosic substrates from stains caused by the rusting of iron objects, both decorative and support, can damage the artworks and often pose a risk to the health of restorers and the environment [48]. In one of our recent works, chitosan-based hydrogels containing acetic, oxalic and citric acids were tested and the chitosan-oxalic acid hydrogel was the most successful for removing rust stains from the surface of different lithotypes by exploiting the reducing capacity of carboxylic acid with the chelating properties of the polysaccharide [49]. In this contest, DESs, thanks to their ability to dissolve metal oxides, could be suitable alternative solvents in removing corrosion products from a valuable surface.

Our objective was to evaluate the efficacy of DESs composed of choline chloride and acetic (ChCl/Ac), oxalic (ChCl/Ox) and citric (ChCl/Cit) acids to dissolve and remove rust stains from cellulose-based and lithic substrates in view of their possible use also on precious materials in the field of cultural heritage.

All the three DESs were previously well characterized; their physicochemical properties and, in some cases, their capability to solubilize metal oxides were already evaluated [40,41,50,51]. Oxalic acid-based DES, due to its good reducing properties, shows the highest dissolution ability of metal oxides even when compared with 1.8 M oxalic acid aqueous solution [45]. Regarding the citric-acid-based DES, its bulky structure produces a highly viscous solvent so that, to decrease the viscosity of the medium and to leach spent lithium-ion batteries, the molar ratio of ChCl/Cit has to be changed from 1:1 to 2:1 with 35% of water added [52]. To our knowledge, none of these DESs were ever employed to remove rust from lithic or cellulosic substrates.

Different techniques were employed to ensure both full rust removal and absence of side effects on the substrate, as macroscopic and microscopic observations, colorimetry and SEM/EDS microanalyses, which allow the identification of the constituent elements (elemental analysis) even present in traces.

## 2. Results and Discussion

In the field of cultural heritage, cleaning of stone surface and cellulosic substrates from metal stains is one of the most challenging issues and DESs could represent an effective and green approach. 

In the following, we report the effectiveness of the three DESs, i.e., ChCl/Ac, ChCl/Ox and ChCl/Cit, toward artificially stained linen canvas, used as painting support since ancient times, and lithic materials, namely granite and travertine, widely present in monumental cultural heritage.

Although the high viscosity of DESs often limits their application in chemical processes, in the field of restoration it can be an added value thanks to a lower tendency to drain if the product is applied on vertical surfaces. The viscosity of the three DESs was measured as a function of temperature and the results are reported in Figure S1. All DESs show an exponential decreasing trend with increasing temperature and the ChCl/Ac is the less viscous (100–50 cP). Viscosity of ChCl/Ox (250–100 cP) results about double that of acetic acid-based DES while that of ChCl/Cit (6000–1000 P) is always three orders of magnitude higher.

### 2.1. Canvas

#### 2.1.1. Photo, Stereomicroscope Observations and Colorimetry

On each of the four well-distinct areas of the linen canvas, a 4 × 4 cm^2^ rusty iron grid was placed to induce staining; then, the effectiveness in removing the corrosion products of the three DESs was compared using water as control. The entire process was replicated twice on two distinct linen canvases to confirm the results.

**Figure 1:**
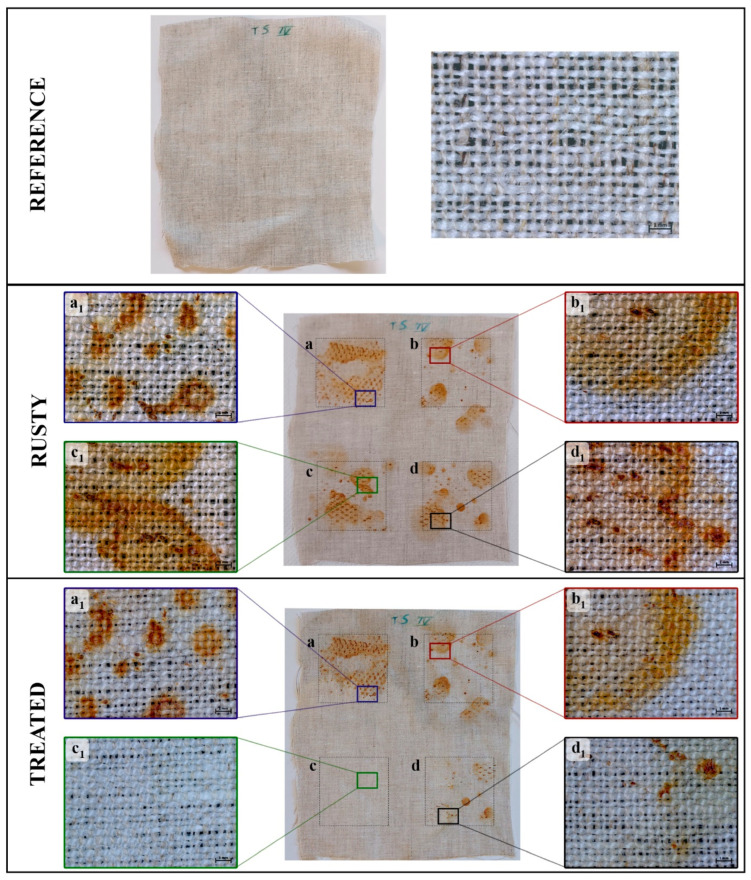
Photographs of a canvas, before (reference) and after the staining process (rusty) and following the treatment (treated) by using water (area **a**), ChCl/Ac, (area **b**) ChCl/Ox (area **c**) and ChCl/Cit (area **d**). For all the rusty and treated areas, the stereomicroscope images of 90 mm^2^ subareas (**a_1_**–**d_1_**) at 12.5× magnification. Space bar = 1 mm.

Figure 1 shows the photos of one of the canvases before and after the staining process and following the treatment with water (area a), ChCl/Ac (area b), ChCl/Ox (area c) and ChCl/Cit (area d) DESs, confined in cellulose poultices. Moreover, in the same a-d areas, choosing the spots among the darkest ones, stereomicroscope images were acquired on subareas of about 90 mm^2^. The images of the four subareas, a_1_–d_1_, rusted and treated are shown in the same Figure 1.

As evident from the photos, the rust is not homogeneously distributed on the selected four areas of canvas, but more or less large orange spots appear randomly. This visual perception is confirmed by the mean colorimetric measurements reported in Table S1. Data were acquired on 16 points of each of the two canvases by using a 4 × 4 grid to cover approximately 60% of the areas a–d. Due to the inhomogeneity of the staining process, the mean differences of the chromatic coordinates corresponding to each rust area are characterized by high standard deviations. 

Regarding the photos of the treated areas reported in Figure 1, following the application of water and ChCl/Ac, no change can be observed, while the ChCl/Cit system seems to be able to remove only the lighter stains, leaving the darker ones unchanged. In contrast, with ChCl/Ox, no trace of rust can be detected in the whole treated area. Despite the high standard deviations of the colorimetric data, in Table S1, the ChCl/Ox treated area results are characterized by a color change, calculated by means of Equation (1), ΔE* < 2 that confirms what has just been stated.

To better compare the ability of DESs in removing the darkest iron stains, colorimetry data (Table 1) was also collected on one of the two linen canvases, by acquiring the measurements in triplicate on each of its subareas a_1_–d_1_ observed by the stereomicroscope.

The chromatic parameters of the stained subareas, when compared with those of the reference, clearly show an increase in yellow and red coordinates as well as a decrease in brightness. Consequently, the ΔE* values of the stained subareas range between 14 and 28. After the application of water and ChCl/Ac, the color parameters in a_1_ and b_1_ subareas remain unchanged, while after the treatment with ChCl/Cit, subarea d_1_, they result about 1.5–2 times lower than their corresponding ones before the treatment. As already anticipated, only the oxalic acid-based DES removes the rust stains completely, its chromaticity being similar to that of the reference. Indeed, in c_1_ treated subarea, the value ΔE* = 1.2 is well below the threshold limit of perception of the human eye (ΔE* < 3) [53].

The efficacy of the ChCl/Ox was then tested on a larger area of a linen fabric stained with a 10 × 10 cm^2^ rusty grid. As evidenced by the colorimetric analysis reported in Table S2, the eutectic mixture conveyed on the large and rusted canvas was confirmed to be effective in removing all the stains. In fact, although the dark orange-stained fabric shows an overall color alteration, ΔE* = 14 ± 8, after the treatment with the ChCl/Ox DES and a carful rinse with wet cellulose poultice, the color difference decreases well below the perception limit of the human eye, ΔE* = 2.0 ± 0.5.

#### 2.1.2. SEM/EDS Analysis

**Figure 2:**
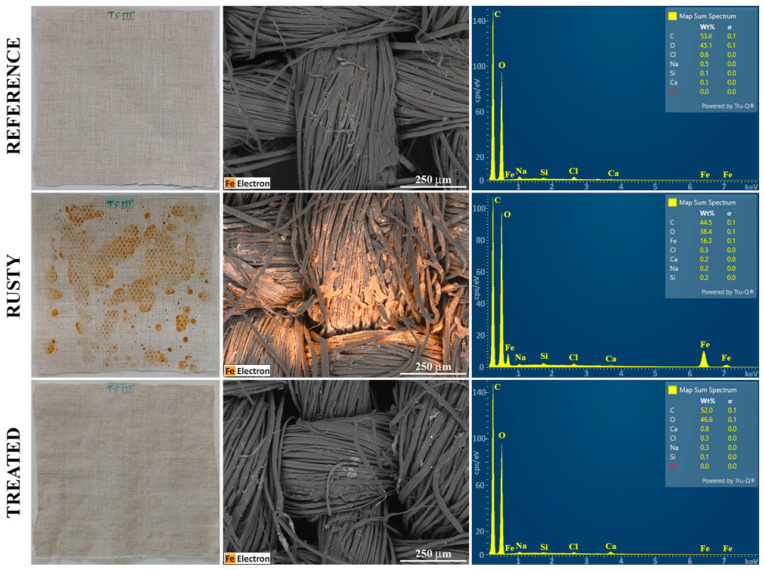
Phases of the experimentation on a large linen canvas: photos (on the **left**), SEM images (in the **middle**), and EDS spectra (on the **right**) of 1 mm^2^ area of the entire surface, before (reference) and after the staining (rusty) and the treatment with ChCl/Ox (treated). In SEM images, the presence of Fe is highlighted in orange.

SEM/EDS analysis is a very sensitive technique capable of detecting elements even in trace amounts; therefore, it was selected to evaluate both the effectiveness of ChCl/Ox in removing iron oxides from rusty linen fabric and the eventual presence of residues. Figure 2 reports photos, SEM images, in which the presence of iron was highlighted in orange, and the corresponding EDS spectra of one of the three 1 mm^2^ areas acquired on the reference large canvas (first row), after the staining (second row) and the treatment (third row) procedures.

The photos in the first column reflect the results shown in Table S2, i.e., an inhomogeneous deposition of the rust products on the stained canvas and the effectiveness of a single treatment with ChCl/Ox confined within the cellulose pulp to restore the original chromaticity of the sample.

The SEM images highlight how, following the staining process, orange-colored crusts appear between the linen fibers, corresponding to the iron element, and how they are totally absent on the treated sample. These images confirm the total removal of rust, not only at a macroscopic level, as observed in the photos, but also at a microscopic one. Furthermore, after the treatment, the morphology of the fabric fibers is very similar compared to the reference, indicating that DES does not alter the substrate.

The mean elemental abundance of the main constituents of the substrate, carbon and oxygen, together with iron, were obtained by carrying out EDS microanalysis on the three selected areas of the reference, stained and treated linen surfaces, and they are shown in Table 2. The corresponding raw data are available in Table S3. 

Being mainly composed by cellulose, the original and uncontaminated linen canvas results, as expected, are almost constituted by carbon and oxygen. Moreover, very low amounts of sodium, silicon, chlorine and other elements are present, while iron does not seem to be a significant impurity included in the linen fiber. On the contrary, the EDS spectra of the stained surface shows a high relative abundance of iron, equal to 16.4% wt, and a consequent decrease in the oxygen and carbon ones. After the treatment with ChCl/Ox, the EDS analysis highlights the complete disappearance of iron, the absence of any other impurities resulting from the treatment and the restoration of the percentages of the main elements characterizing the reference surface. In addition, the EDS spectra of the treated area show a similar content of chlorine compared to the reference and stained ones (Figure 2, third row), clearly indicating the effective removal of the DES from the fabric.

### 2.2. Stone

#### 2.2.1. Photo and Colorimetry 

**Figure 3:**
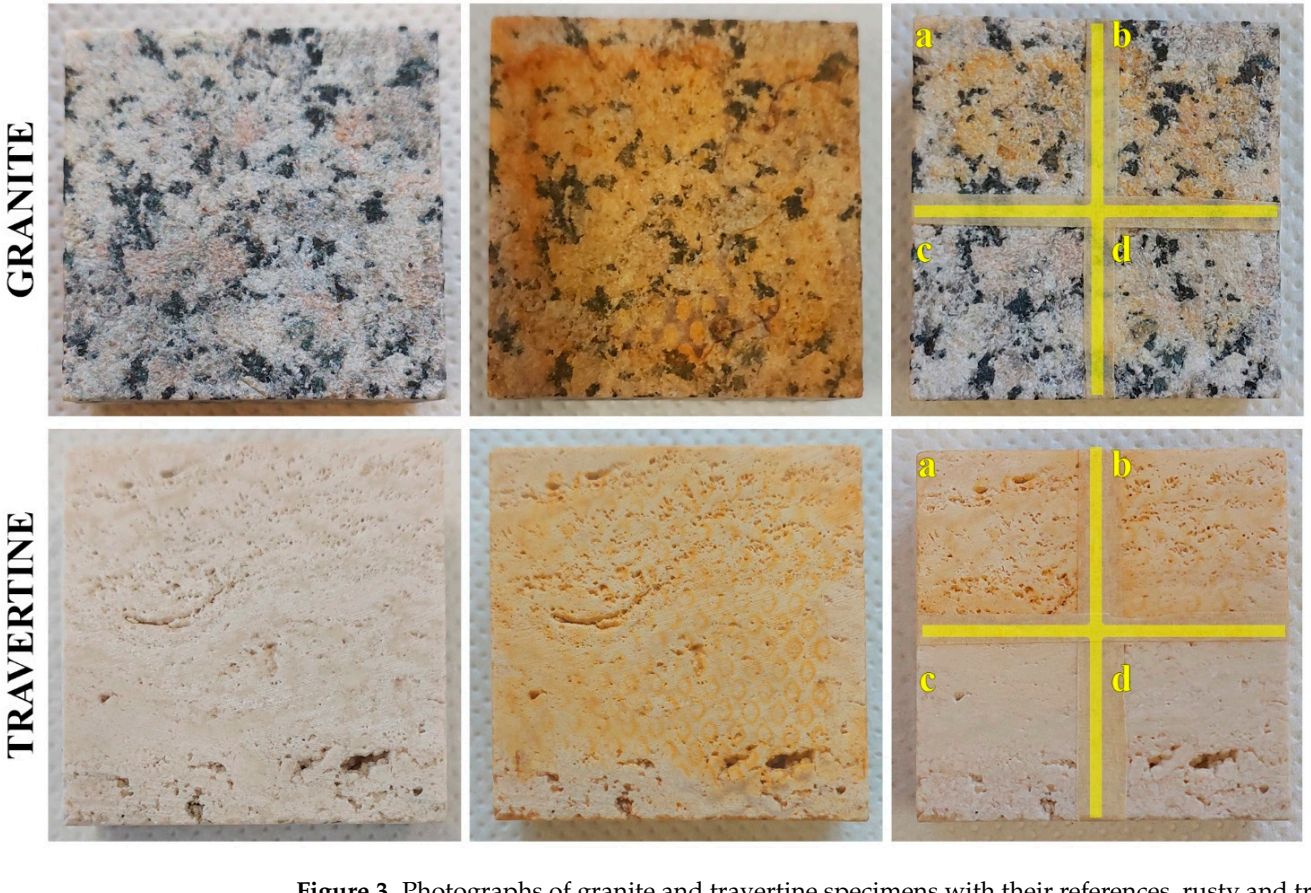
Photographs of granite and travertine specimens with their references, rusty and treated areas by using water (**a**), ChCl/Ac (**b**), ChCl/Ox (**c**) and ChCl/Cit (**d**).

For both granite and travertine lithotypes, differing in composition and in open porosity, two samples were stained and divided into four 2 × 2 cm^2^ areas (a–d) with an adhesive paper strip, to compare the effectiveness of the different treatments. Water as control (area a), ChCl/Ac (area b), ChCl/Ox (area c) and ChCl/Cit (area d) were applied with a cotton swab and removed after 15 min. Figure 3 reports the photos in the various phases of the experimentation on one of the two used specimens for each lithotype.

Compared to the canvas, a homogeneous layer of rust was deposited on both the stones. After the cleaning procedure, evident rust residues remain on the areas treated with water (a) and ChCl/Ac (b), clearly indicating that the removal of the corrosion products was achieved only for the rust not well adhered on the specimen surfaces. On the contrary, the areas of granite and travertine treated with the ChCl/Ox (c) and ChCl/Cit (d) resulted as almost completely cleaned. However, on the granite, the silicate-based substrate, a slight residual yellowish color is detectable on the area d, treated with citric acid-based DES.

To evaluate the effective restoration of the original hue for both lithotypes, the measurements of colorimetric coordinates were replicated on two samples each and the mean values are listed in Table 3.

The deposition of rust affected the chromaticity of the lithic substrates, decreasing their brightness (negative value of ΔL*) and increasing both the red and yellow coordinates (positive values of Δa* and Δb*). On granite, the application of water (area a) and ChCl/Ac (area b) seems to remove more rust than that on travertine, probably due to its lower porosity, which can limit the adhesion of the rust particles on the stone surface. However, for both lithotypes, the color alteration (ΔE* ~ 15) is higher than that considered acceptable after a restoration intervention (ΔE* < 5) [53].

In contrast, the treatments with ChCl/Ox (area c) and ChCl/Cit (area d) on both the lithic substrates induced an almost complete restoration of the original chromaticity, their ΔE* always being lower than 4.

To test the cleaning procedure on larger surfaces, two granite and travertine 5 × 5 × 2 cm^3^ samples were stained with the rusty iron grid. Since color differences after the treatment with ChCl/Ox and ChCl/Cit were both acceptable, ChCl/Cit was selected as the cleaning product, for its higher viscosity, a feature that facilitates the application of the product for restoration works even on vertical surfaces.

Colorimetry results, shown in Table S4, confirm the good performance of the chosen DES. In fact, after the treatment with ChCl/Cit, the almost complete restoration of the stone chromaticity was achieved for granite as well as travertine, with their ΔE*s equal to 1.2 and 3.1, respectively.

#### 2.2.2. SEM/EDS Analysis

**Figure 4:**
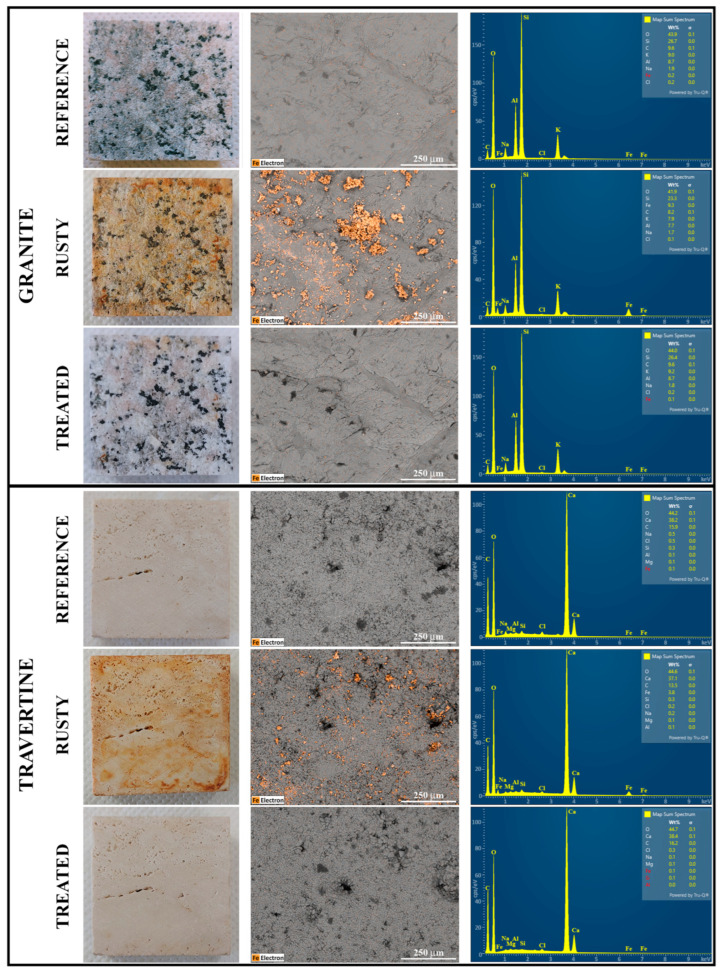
Phases of the experimentation on granite and travertine specimens: photos (on the **left**), SEM images (in the **middle**), and EDS spectra (on the **right**) of reference, rusty and treated stone surfaces. In SEM images, the presence of Fe is highlighted in orange.

For both lithotypes, SEM/EDS analysis was carried out before and after the staining as well as following the treatment on one of the two 5 × 5 cm^2^ surfaces with ChCl/Cit. In this way, it was possible to quantify the iron contents before and after the staining and the traces of iron, eventually present, after the cleaning procedure. Figure 4 shows on the left, the photos of reference, rusty and treated 5 × 5 cm^2^ surfaces, in the middle, the SEM images of the selected 1 mm^2^ surfaces and on the right, the corresponding EDS spectra. In the grey-scale of SEM images, the color orange was assigned to Fe to highlight its presence.

In the SEM images of the rusted surface of both lithotypes, visible changes of the iron content are evident by comparing them with the corresponding reference surfaces. However, the orange spots, indicating the presence of Fe, are larger on the silicate-based stone. These results are quantified through the EDS analysis. In addition to iron, two other main elements were selected for each lithotype: silicon and aluminum for granite, which is characterized by a high content of aluminosilicates and, calcium and carbon for travertine, which is almost completely composed by calcium carbonate. Table 4 reports their mean percentages computed on the samples in three selected areas of their reference, rusty and treated surfaces. The raw data are shown in Table S5. 

The elemental analysis, for the granite and travertine stones, indicates a significant increase in the iron percentages after the staining process, being 8% and 5%, respectively. After only one treatment with ChCl/Cit, regardless of the lithotype composition and morphology, the percentages of iron decrease, returning to the proper values of the reference surfaces.

## 3. Materials and Methods

### 3.1. Materials

All chemicals, choline chloride, glacial acetic acid, oxalic acid dihydrate and citric acid monohydrate were purchased from Sigma Aldrich (St. Louis, MO, USA) and used as received. Cellulose pulp having a fiber length of 200 μm and “*Mantegna*” canvas of 183 g/m^2^, made of 100% crude boiled linen yarn, a cellulose-based material selected as fabric substrate, were supplied by I.M.A.R. Italia s.r.l. (Rome, Italy).

Intrusive silicate and sedimentary carbonate lithotypes, namely rosa beta granite and travertine, were selected as stone substrates and their mean composition and structural features were previously described [54]. Cut samples of 5 × 5 × 2 cm^3^ were purchased from Elia Marmi S.n.c. L’Aquila (AQ), L’Aquila, Italy.

### 3.2. Staining of Canvas and Stone

Iron grids were immersed in a 1 M NaCl aqueous solution for one month to accelerate their corrosion. Three 15 × 15 cm^2^ pieces of linen canvas were put in contact with the rusty iron elements and placed between several wet sheets of filter paper to favor the rust contamination.

Four stone specimens for each lithotype were stained by placing the rusty iron grids on one of their 5 × 5 cm^2^ surfaces and by submerging them in distilled water to favor the deposition of the corrosion products following the procedure previously developed and described [49]. 

### 3.3. DES Preparation

Three different DESs were prepared by combining choline chloride, the hydrogen bond acceptor, with acetic (ChCl/Ac), oxalic (ChCl/Ox) or citric (ChCl/Cit) acid, the hydrogen bond donor, in the mole ratio of 1:2, 1:1 and 1:1, respectively [40,41,50,51].

The three mixtures were heated at 70 °C for the required minimum time to obtain homogeneous and transparent liquids and stored at room temperature to avoid the esterification reaction between their constituents [55]. The viscosity of the three DESs was measured by means of Fungilab Viscolead mod. ADV L rotational viscosimeter, in a temperature range between 20 °C and 40 °C. Table S6 shows the RPM and the corresponding spindle used for viscosity determination at each temperature.

### 3.4. DES Application

For linen fabric, each of the eutectic solvent or water, used as control, was confined inside cellulose poultice to slow down its absorption on the substrate. In particular, mixture of 3 g of DES per gram of cellulose poultice was prepared and applied (~70 mg/cm^2^). Each treatment was kept acting for 1 h before its removal from the fabric surface. Then, two subsequent rinses (6 g of water *per* gram of cellulose poultice), lasting 30 min each, were carried out using wet poultice.

The stones were treated differently; the eutectic solvents (~12 mg/cm^2^) or water were applied on the stone surface with the aid of a cotton swab and kept for 15 min. Then, the surface was rinsed with distilled water and gently brushed to remove all the by-products.

### 3.5. Colorimetry

Colorimetric measurements were performed using a portable colorimeter Sama Tools SA230 set up in SCE mode with 8° standard observer angle and D65 light with temperature of 6504 K (average daylight, including the UV region). Chromatic coordinates L*, a* and b* were acquired in the CIELAB color space, proposed in 1976 by the International Lighting Commission (CIE) [56], on reference (sample before rust contamination), stained and treated samples, to cover about 60% of the total area of each specimen. Color alteration value, expressed as ΔE*, was computed as a vector sum by using Equation (1),

(1) $$\Delta E^{*}=\sqrt{\Delta L^{*2}+\Delta a^{*2}+\Delta b^{*2}}$$

where ΔL*, Δa* and Δb* represent the changes in the chromatic coordinates of the stained or treated surface and the reference.

### 3.6. Stereomicroscope Observations

Microscope images of reference, rusty and treated linen fabric specimens were acquired by using an AxioZoom V16 (Zeiss, Jena, Germany) stereomicroscope to observe the absorption of the rust products on the fibers and evaluate their removal after the treatment. For each selected area (~16 cm^2^) of the substrate, images, at different magnifications, were collected and elaborated with the Zen Blue 3.3 software. 

### 3.7. SEM-EDS Analysis

SEM-EDS was performed on specimens of the linen fabric and of both the lithotypes to evaluate their iron contents before and after the staining and then, after the cleaning procedure. The acquisitions were carried out in variable pressure mode (VP) with an accelerating voltage of 15 keV and at working distance of 8.5 mm by means of Zeiss GeminiSEM 500 (Zeiss, Jena, Germany) equipped with EDS OXFORD Aztec Energy with INCA X-ACT detector.

Three areas (~1 mm^2^ each) of the examined surface were randomly selected to collect the SEM images and acquire the EDS spectra. Sample relative positions on the slab were saved to correlate the changes in elemental composition during the phases of the experimentation.

## 4. Conclusions

The ability of choline chloride-based DESs to dissolve and remove rust stains from cellulose-based and lithic substrates was explored in view of their possible use also on precious materials in the field of cultural heritage. For this purpose, we chose three known DESs, consisting of choline chloride and acetic, oxalic or citric acid, since carboxylic acids are capable of reducing ferric oxides in more soluble ferrous compounds. The use of acidic aqueous solutions on precious surfaces, like those of art objects, should be avoided as their application and removal would be difficult and even dangerous for the state of conservation of the artifact. Indeed, the fast adsorption of aqueous solutions into the substrate, without a suitable solvating agent, would make it difficult to effectively remove both unreacted acid and by-products. A simple and fast protocol was developed exploiting the high viscous network of the DES, which limits the penetration of the acid into the substrate, confining it mainly on the material surface, and facilitates its application even on vertical surfaces. A 15 min treatment with ChCl/Ox or ChCl/Cit or 2 h treatment with ChCl/Ox were sufficient to completely remove rust from the lithic and cellulosic substrates, respectively. Photo, colorimetry and mainly SEM-EDS analysis not only confirmed the complete removal of all rust products and the absence of any unwanted residues, but also ensured the integrity of the substrate after the treatment, a basic requirement for their application in real case studies.

## Figures and Tables

**Table 1:** Mean colorimetric coordinates on one canvas in triple acquisition, before the staining (reference) and their mean differences (**∆**L*, **∆**a*, **∆**b*) in all subareas reported in Figure 1, rusty and treated by using water (a_1_), ChCl/Ac (b_1_), ChCl/Ox (c_1_) and ChCl/Cit (d_1_). In the last column, the corresponding mean color differences (**∆**E*).

		L*	a*	b*	
	**REF**	80 ± 1	2.6 ± 0.1	5.8 ± 0.3	
**RUSTY**		∆L*	∆a*	∆b*	∆E*
	**a_1_**	−10.9 ± 0.1	3.46 ± 0.01	7.40 ± 0.02	13.6 ± 0.1
	**b_1_**	−13.8 ± 0.2	7.4 ± 0.1	16.0 ± 0.1	22.4 ± 0.3
	**c_1_**	−15.2 ± 0.3	7.8 ± 0.2	14.7 ± 0.2	22.5 ± 0.4
	**d_1_**	−18.1 ± 0.3	9.98 ± 0.01	18.64 ± 0.05	27.8 ± 0.2
**TREATED**		∆L*	∆a*	∆b*	∆E*
	**a_1_**	−11.1 ± 0.1	3.61 ± 0.02	7.0 ± 0.1	13.6 ± 0.1
	**b_1_**	−12.4 ± 0.1	7.26 ± 0.01	17.48 ± 0.03	22.65 ± 0.06
	**c_1_**	0.9 ± 0.5	0.54 ± 0.02	−0.54 ± 0.04	1.2 ± 0.4
	**d_1_**	−8.6 ± 0.3	4.71 ± 0.02	11.71 ± 0.02	15.3 ± 0.2

**Table 2:** From EDS analysis, mean percentages of carbon, oxygen and iron, computed on three selected areas of the reference, rusty and treated surfaces of the large linen canvas.

	Weight, %		
Element	Ref	Rusty	Treated
**C**	53.1 ± 0.4	44 ± 3	51.9 ± 0.2
**O**	45.7 ± 0.7	40 ± 2	46.9 ± 0.5
**Fe**	0.0 ± 0.1	16 ± 4	0.0 ± 0.1

**Table 3:** For each lithotype, mean colorimetric coordinates on two samples, before the staining (reference) and their mean differences in all areas reported in Figure 2, rusty and treated by using water (a), ChCl/Ac (b), ChCl/Ox (c) and ChCl/Cit (d). In the last column, the corresponding mean color differences.

		GRANITE				TRAVERTINE			
		L*	a*	b*		L*	a*	b*	
	**REF**	69 ± 2	0.1 ± 0.7	2 ± 1		80 ± 2	3.8 ± 0.3	10 ± 1	
**RUSTY**		∆L*	∆a*	∆b*	∆E*	∆L*	∆a*	∆b*	∆E*
	**a**	−11 ± 5	11 ± 5	27 ± 6	32	−7 ± 1	7 ± 2	19 ± 4	21
	**b**	−8 ± 3	8 ± 2	22 ± 4	25	−8 ± 2	8 ± 1	23 ± 1	25
	**c**	−6 ± 2	8 ± 2	22 ± 3	24	−7 ± 3	8 ± 2	22 ± 8	25
	**d**	−8 ± 5	9 ± 3	22 ± 6	32	−8 ± 1	8 ± 1	23 ± 4	26
**TREATED**		∆L*	∆a*	∆b*	∆E*	∆L*	∆a*	∆b*	∆E*
	**a**	−5 ± 2	3 ± 3	9 ± 7	11	−5 ± 1	5.1 ± 0.7	14 ± 3	15
	**b**	−2 ± 2	2 ± 1	6 ± 2	7	−6 ± 1	5.9 ± 0.6	15 ± 2	17
	**c**	2 ± 2	0.1 ± 0.3	−1.3 ± 0.8	2.0	0 ± 1	1.1 ± 0.3	2.9 ± 0.8	3.1
	**d**	−2 ± 2	1.3 ± 0.9	3 ± 3	3.7	0 ± 1	1.0 ± 0.4	2.2 ± 0.4	2.5

**Table 4:** From the EDS analysis, mean percentages of the most significant elements, including Fe, of travertine and granite, determined in three areas of their reference, rusty and treated surfaces.

		Weight, %		
	Element	Ref	Rusty	Treated
**Granite**	**Si**	28 ± 5	26 ± 5	29 ± 6
	**Al**	7 ± 3	6 ± 3	7 ± 3
	**Fe**	1 ± 1	8 ± 1	1 ± 1
**Travertine**	**Ca**	36 ± 2	36 ± 1	36 ± 2
	**C**	17 ± 1	13.8 ± 0.4	20 ± 3
	**Fe**	0.1 ± 0.1	5 ± 1	0.1 ± 0.1

## Data Availability

Data are contained within the article and Supplementary Materials.

## Supplementary Materials

The following supporting information can be downloaded at: https://www.mdpi.com/article/10.3390/molecules29030624/s1. Figure S1: Viscosity of ChCl/Ac, ChCl/Ox and ChCl/Cit as a function of temperature. Table S1: Mean colorimetric coordinates on 16 points of each of the two canvases, before the staining (reference) and the mean differences (∆L*, ∆a*, ∆b*) in all areas reported in Figure 1, rusty and treated with water (a), ChCl/Ac (b), ChCl/Ox (c) and ChCl/Cit (d). In the last column, the corresponding mean color differences (∆E*). Table S2: Mean colorimetric coordinates acquired on a large linen canvas before the staining (reference) and their mean differences (∆L*, ∆a*, ∆b*) in the entire surfaces, rusty and treated by using ChCl/Ox. In the last column, the corresponding mean color differences (∆E*). Table S3: From the EDS analysis, row data of the percentages of carbon, oxygen and iron, in three selected areas of the reference, rusty and treated surfaces of the large linen canvas. Table S4: For each lithotype, mean chromatic coordinates of two stones, before the staining (reference) and their mean differences in the entire surfaces, rusty and treated by using ChCl/Cit. In the last column, the corresponding mean color differences (∆E*). Table S5: From EDS analysis, raw data of the percentages of the most significant elements of travertine and granite, including iron, acquired on two samples each, in three areas of their reference, rusty and treated surfaces. Table S6: Revolution per minutes (RPM) to determine the viscosity of the three DESs as a function of temperature. Spindle L1 was used for ChCl/Ac and ChCl/Ox, while spindle L4 was used for ChCl/Cit.
