# Supplementary material for: Natural Deep Eutectic Solvents as Rust Removal Agents from Lithic and Cellulosic Substrates
Source: Molecules. 2024 Jan 28;29(3):624. doi: 10.3390/molecules29030624 (PMC10856158; doi:10.3390/molecules29030624)
Supplement: Supplementary file 1 [file molecules-29-00624-s001.zip › molecules-2793529-supplementary.pdf]

SUPPLEMENTARY MATERIALS

NATURAL DEEP EUTECTIC SOLVENTS AS RUST REMOVAL AGENTS FROM LITHIC AND CELLULOSIC SUBSTRATES

Francesco Gabriele, Cinzia Casieri and Nicoletta Spreti*

Department of Physical and Chemical Sciences, University of L'Aquila, Coppito, I-67100 L'Aquila, Italy

*Corresponding author: nicoletta.spreti@univaq.it

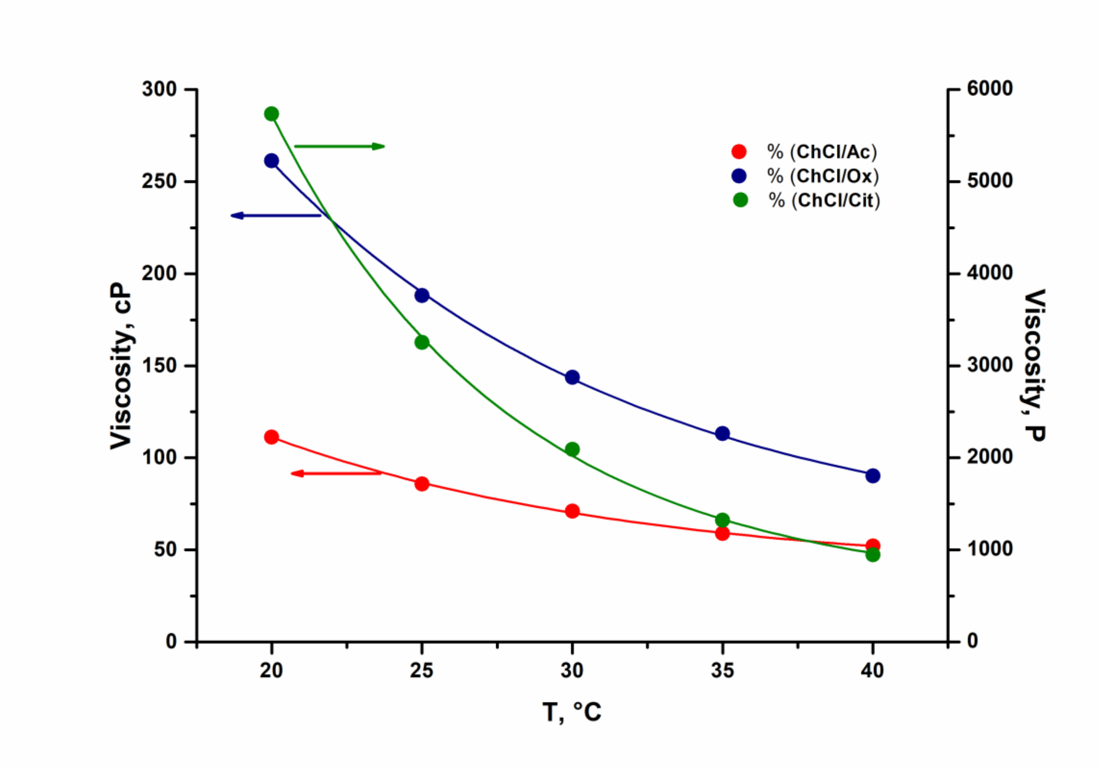

Figure S1. Viscosity of ChCl/Ac, ChCl/Ox and ChCl/Cit as a function of temperature.

Table S1. Mean colorimetric coordinates on 16 points of each of the two canvases, before the staining (reference) and the mean differences (ΔL*, Δa*, Δb*) in all areas reported in Figure 1, rusty and treated with water (a), ChCl/Ac (b), ChCl/Ox (c) and ChCl/Cit (d). In the last column, the corresponding mean color differences (ΔE*).

		**L***	**a***	**b***	
	**REF**	80±1	2.6±0.1	5.8±0.3	
		**ΔL***	**Δa***	**Δb***	**ΔE***
**RUSTY**	**a**	-12±4	5±3	12±5	18±7
	**b**	-6±6	2±4	4±7	8±9
	**c**	-10±5	4±3	9±6	14±8
	**d**	-8±5	3±3	7±6	11±8
		**ΔL***	**Δa***	**Δb***	**ΔE***
**TREATED**	**a**	-11±4	5±3	11±6	18±7
	**b**	-4±5	2±2	3±6	6±8
	**c**	0.9±0.5	-0.3±0.1	-0.9±0.5	1.4±0.5
	**d**	-2±4	2±2	5±5	6±6

Table S2. Mean colorimetric coordinates acquired on a large linen canvas before the staining (reference) and their mean differences (ΔL*, Δa*, Δb*) in the entire surfaces, rusty and treated by using ChCl/Ox. In the last column, the corresponding mean color differences (ΔE*).

	**L***	**a***	**b***	
**REF**	80±1	2.6±0.1	5.8±0.3	
	**ΔL***	**Δa***	**Δb***	**ΔE***
**Rusty**	-11±5	4±3	8±7	14±8
**Treated**	0±1	-0.4±0.2	1.6±0.4	2.0±0.5

Table S3. From the EDS analysis, row data of the percentages of carbon, oxygen and iron, in three selected areas of the reference, rusty and treated surfaces of the large linen canvas.

	**Weight %**								
	**Reference**			**Rusty**			**Treated**		
	**Area 1**	**Area 2**	**Area 3**	**Area 1**	**Area 2**	**Area 3**	**Area 1**	**Area 2**	**Area 3**
**C**	52.8	53.5	52.9	44.5	40.1	46.4	51.9	51.8	52.1
**O**	46.4	45.0	45.8	38.5	39.5	41.6	46.7	47.5	46.6
**Fe**	0.0	0.0	0.1	16.4	19.4	11.5	0.0	0.0	0.1

Table S4. For each lithotype, mean chromatic coordinates of two stones, before the staining (reference) and their mean differences in the entire surfaces, rusty and treated by using ChCl/Cit. In the last column, the corresponding mean color differences (ΔE*).

	GRANITE				TRAVERTINE			
	**L***	**a***	**b***		**L***	**a***	**b***	
**REF**	70±3	0±0.5	2.0±0.8		80±1	3.5±0.3	8.3±0.9	
	**ΔL***	**Δa***	**Δb***	**ΔE***	**ΔL***	**Δa***	**Δb***	**ΔE***
**Rusty**	-4±3	6±2	18±3	19	-6±2	6±2	15±3	18
**Treated**	1±2	0.2±0.8	0.6±0.2	1.2	1±1	0.5±0.5	3±1	3.1

Table S5. From EDS analysis, raw data of the percentages of the most significant elements of travertine and granite, including iron, acquired on two samples each, in three areas of their reference, rusty and treated surfaces.

		Weight %								
		**Reference**			**Rusty**			**Treated**		
		**Area 1**	**Area 2**	**Area 3**	**Area 1**	**Area 2**	**Area 3**	**Area 1**	**Area 2**	**Area 3**
**Granite**	**Si**	26.2	34.2	24.5	22.8	31.1	22.5	26.4	35.7	24.8
	**Al**	8.6	3.2	8.6	7.5	2.9	8.0	8.7	2.8	8.8
	**Fe**	0.2	0.4	2.7	9.1	7.3	8.1	0.1	0.1	2.2
**Travertine**	**Ca**	38.3	36.2	34.2	37.1	35.3	36.1	38.3	34.2	34.3
	**C**	15.6	16.9	17.5	13.5	13.7	14.2	16.3	21.5	21.1
	**Fe**	0.1	0.2	0.1	3.8	5.6	4.0	0.1	0.0	0.1

Table S6. Revolution per minutes (RPM) to determine the viscosity of the three DESs as a function of temperature. Spindle L1 was used for ChCl/Ac and ChCl/Ox, while spindle L4 was used for ChCl/Cit.

**T, ° C**	**RPM**		
	**ChCl/Ac**	**ChCl/Ox**	**ChCl/Cit**
20	50	20	1
25	60	30	1.5
30	60	30	2.5
35	60	50	4
40	100	60	6
